# Characterization of the Cultivable Endophytic Bacterial Community of Seeds and Sprouts of *Cannabis sativa* L. and Perspectives for the Application as Biostimulants

**DOI:** 10.3390/microorganisms10091742

**Published:** 2022-08-29

**Authors:** Morena Gabriele, Francesco Vitali, Elisa Chelucci, Carolina Chiellini

**Affiliations:** 1 Institute of Agricultural Biology and Biotechnology, Italian National Research Council, Via Moruzzi 1, 56124 Pisa, Italy; 2 CREA Council for Agricultural Research and Analysis of the Agricultural Economy, Research Centre for Agriculture and Environment, Via di Lanciola n.12/a, Cascine del Riccio, 50125 Florence, Italy

**Keywords:** *Cannabis sativa*, bacterial endophytes, seeds, indole-3-acetic acid, stress resistance, polyphenols, antioxidant activity

## Abstract

Endophytes are beneficial microorganisms exerting growth-promoting activities in plants; they are most often located within the plant intercellular spaces and can be found in all plant tissues, including roots, leaves, stems, flowers, and seeds. In this work, we investigated the cultivable bacterial community of the seeds and the two-week sprouts of the *Cannabis sativa* L. cultivar “Futura 75”. Endophytes were genotypically and phenotypically characterized and were exposed to different concentrations of seed extracts to verify their susceptibility. A bacterial strain among all the isolates was selected for germination tests of *C. sativa* in different experimental conditions. The results revealed the dominance of *Firmicutes* (*Staphylococcus* sp.) among the isolated strains. Two strains were different from the others for indole-3-acetic acid (IAA) production and for their resistance patterns towards abiotic and biotic stresses. The *Sphingomonas* sp. strain Can_S11 (*Alphaproteobacteria*) showed a potential ability to increase the nutraceutical features of its sprouts, particularly an increase in the polyphenol content and antioxidant activity. None of the isolated strains were susceptible to the seed extracts, which were previously tested as antimicrobial and antibiofilm agents against human pathogenic bacteria. The results open new perspectives for the study of the endophytes of *C. sativa* as possible biostimulants.

## 1. Introduction

Endophytes include beneficial microorganisms exerting well-known growth-promoting activities in plants, such as seed germination, growth support, nutrient supply, stimulation of metabolite production, and resistance against biotic and abiotic stresses [1]. The selection and shaping of the endophytic community in a plant mainly depend on specific factors, including the characteristics of the microbial population, the plant genotype, and other environmental factors such as the soil type [2]. Interestingly, endophytes can affect the plant phenotype, determining its phytochemical profile and mediating the expression of the plant’s functional traits [3].

Bacterial endophytes are most often located within the plant’s intercellular spaces, which are rich in carbohydrates, amino acids, and inorganic nutrients and can be found in all the plant tissues including the roots, leaves, stems, flowers, and seeds [4,5]. Seed endophytes are transmitted from generation to generation, assuring their presence in new plants; this is defined as vertical transmission, referring to direct transfer from parent to progeny [6].

Seed endophytes might exit the seed and enter in the plant through, for example, the plant surface, or they might remain inside the seed and be spread through plant growth or move within the plant tissue [7]. Seed-borne bacteria have been described for their growth-promoting activity on seedlings, which is exerted by hormone production and modulation [8,9]. Moreover, seed bacterial endophytes showing plant-growth-promoting (PGP) traits, such as the ability to produce indole-3-acetic acid (IAA) or 1-aminocyclopropane-1-carboxylate (ACC) deaminase, were used as an inoculum for plants, resulting in the improvement of plant growth compared with the non-inoculated controls [10,11].

Hemp (*Cannabis sativa* L., Cannabaceae family) is an annual herbaceous plant that originated in Central Asia and was historically used for medical treatments and as a food and fiber source. Among the natural compounds characterizing the phytochemistry of *Cannabis sativa* are cannabinoids, in particular Δ9-tetrahydrocannabinol (THC). This psychoactive compound is responsible for the intoxicant properties of the cannabis plant, and its presence differentiates between ‘hemp’ (non-drug) and ‘marijuana’ (drug type). The drug type, *C. sativa* subsp. indica, can contain up to 20% THC, whereas the non-drug and industrial type, *C. sativa* subsp. sativa, is characterized by a low content of THC [12,13]. Currently, hemp seeds are mainly used as animal feed, and among their products, the oil, meal, flour, and protein powder represent important sources of essential amino acids and fatty acids for human nutrition [12,13]. In addition, hemp seeds and sprouts, being rich in phytochemical compounds, especially polyphenols (caffeoyltyramine and cannabisin A, B, and C), amino acids, and saccharides, represent promising functional foods [13].

Among the endophytes of *Cannabis sativa*, many fungi have been described, especially in relation to their ability to produce secondary metabolites with antifungal activity against phytopathogens [14,15]. More recently, bacterial endophytes of *C. sativa* were also investigated for their antifungal activity [16]. Up to now, the literature concerning the genotypic and phenotypic characterization of the endophytic community of *C. sativa* is still scanty, especially the literature related to the seeds and the cultivable fraction of bacteria. Interestingly, the biostimulant activity of hemp endophytes was reviewed, especially regarding their ability to trigger the production of IAA-like molecules in the *C. sativa* plant [17].

In recent years, sprouted seeds at very young growth stages are gaining great attention as ready-to-eat vegetable products and are widely and regularly consumed as sources of functional and bioactive molecules, including vitamins, essential amino acids, γ-aminobutyric acid, soluble fiber, phenolic acids, flavonoids, selenium-containing compounds, etc., which can exert health-promoting effects [18].

In this work, we investigated the cultivable bacterial community of the seeds and two-week-old sprouts grown in vitro of the *Cannabis sativa* L. cultivar “Futura 75”. Both the taxonomic attribution and the phenotypic characterization (biotic and abiotic stress resistance patterns and IAA production) were evaluated. Isolated endophytes were exposed to different concentrations of seed extracts that were previously tested against human pathogenic bacteria [19] in order to verify their eventual susceptibility. Finally, a bacterial strain among all the isolated endophytes was selected for preliminary germination tests of *C. sativa* in different experimental conditions, opening possible perspectives for a more extended study of the endophytes of *C. sativa* as possible biostimulants.

## 2. Materials and Methods

### 2.1. Plant Material and Seed Extraction

The seeds of *Cannabis sativa* L. cultivar Futura 75, provided by ASSOCANAPA (Carmagnola, Turin, Italy), were extracted according to Frassinetti et al. [13]. Briefly, 1 g of *C. sativa* seeds in 10 mL of 80% ethanol were homogenized with an Ultraturrax (Kinematica Polytron PT MR 2100, Eschbach, Germany) and extracted in the dark for 3 h while shaking. After 20 min of centrifugation (Jouan CR 31 centrifuge, Newport Pagnell, UK) at 4500× *g*, the supernatant was collected, filtered (0.2 μm VWR International PBI, Milan, IT), and kept at −20 °C in the dark until use.

### 2.2. Isolation of Bacterial Strains from Seeds

A total of 60 seeds were surface-sterilized in a 50 mL sterile falcon tube following the protocol described in Scott et al. [16], which was modified as follows: 1 min 70% ethanol, 3 min 3% hypochlorite, 30 s 70% ethanol, 3 min sterile distilled water, and 2 min sterile distilled water (second turn). An aliquot of the distilled water from the last washing was plated in triplicate in solid Tryptone Soy Agar (TSA) medium to verify the absence of any external contamination (e.g., bacterial epiphytes) at the end of the sterilization process. Sterilized seeds were split into two falcon tubes: in the first one, 30 sterilized intact seeds were placed together with 15 mL of sterile Tryptone Soy Broth (TSB) medium; in the second one, 30 seeds were placed, after their disruption, in a sterile mortar with 15 mL of sterile TSB medium. Hence, the two falcon tubes, respectively containing intact seeds (IS) and disrupted seeds (DS), were placed at 24 °C for 1 week in static conditions. A negative control represented by the TSB medium was also added (15 mL) to verify the absence of contamination during the 7 days. After one week, and after verifying the absence of external contamination from both the TSA plates with the water of the last washing and the TSB control of the falcon tubes, we plated in triplicate 100 µL of the “enriched” medium from both the IS and DS samples, and we incubated the plates at 24 °C for 1 week, monitoring the growth of bacterial colonies after 48, 72, and 120 h and after 7 days. During the 7 days of growth, different bacterial colonies from the plates were randomly chosen, picked up, and streaked into TSA plates to isolate them in pure cultures. The isolation in pure cultures occurred after three passages in TSA plates. Finally, the isolated strains were cryopreserved in 20% glycerol and stored at −80 °C in the laboratory collection of the Institute of Agricultural Biology and Biotechnology of the Italian National Research Council in Pisa.

### 2.3. Isolation of Bacterial Strains from Sprouts

A total of 30 additional sterilized seeds were used for germination under in vitro growth. Seeds were placed in Petri dishes (10 seeds for each plate) containing sterile water supplemented with 0.7% agar (Merck Millipore, Darmstadt, Germany) and kept for two weeks in the growth chamber under a controlled temperature (24–22 °C) and a 16/08 h day–night cycle with a Photosynthetic Photon Flux Density (PPFD) of 70 µmol photons m^−1^ s^−1^. After two weeks, no contamination was present on the surfaces of the agar plates (e.g., no mold and/or bacterial growth). Then, 20 sprouts were randomly chosen in sterile conditions and subdivided into two subsets: the first 10 sprouts were put in a sterile 50 mL falcon tube together with 10 mL of TSB medium (sample “GI”), while the second 10 sprouts were disrupted in a sterile mortar and the pieces were put in a second sterile 50 mL falcon tube with 10 mL of TSB medium (sample “GR”). These two treatments, hereafter called “enrichments”, were kept for 1 week in agitation at 24 °C, together with a negative control represented by the non-inoculated TSB medium. At the end of the aforementioned experimental period, the negative control was limpid, and no bacterial growth was observed, whereas in the two enrichment treatments, we observed microbial growth. For each treatment, we plated 100 µL of inoculum in TSA plates in triplicate, for a total of six plates (three from GI enrichment and three from GR enrichment). The plates were incubated at the same temperature as the inoculum, and after 1 week, bacterial colonies had grown on the surface of the agar medium. Since we observed unique morphologies in all six plates, we isolated six strains from GI enrichment (isolates GI_1 to GI_6) and six strains from GR enrichment (isolates GR_1 to GR_6), according to the procedure described in the previous paragraph.

### 2.4. Molecular Characterization of the Isolated Strains and Phylogenetic Analysis

A total of 24 bacterial strains isolated from the seeds and a total of 12 strains isolated from the enriched samples were analyzed through ARDRA screening, as described in Chiellini et al. [20]. Briefly, 16S rRNA was amplified in a 15 µL reaction from the bacterial cell lysates obtained from isolated bacterial colonies grown forb48 h in TSA medium using primers F7 (5′-AGAGTTTGATYMTGGCTCAG-3′) and R1492 (5′-GGNWACCTTGTTACGACTT-3′), as described in Chiellini et al. [21]. Thermocycling was performed using a Labnet MultiGene™ Gradient PCR Thermal Cycler. The 16S rRNA gene-sized fragments were then digested with the Fast Digest BsuRI restriction enzyme (Thermo fisher Scientific, Walthamm, MA, USA), following the protocol suggested by the manufacturer. Digested fragments were visualized by electrophoresis on 2% agarose gel. Fragments showing identical electrophoresis patterns (haplotype) were grouped together. One strain for each identified haplotype was chosen for the molecular characterization through 16S rRNA amplification and sequencing. Then, 16S amplification was conducted, as described above, in a total volume of 30 µL. The obtained amplicons were purified through ethanol precipitation and sent to the Mycrosynth company (Germany) for sequencing. The obtained sequences were processed through the NCBI Blast tool [22] to determinate their preliminary affiliation by comparing the sequences with all the sequences present in the international databases. The phylogenetic analysis was conducted with the maximum-likelihood method on a total of 110 sequences (96 high-quality sequences selected from international databases and 14 sequences belonging to our endophytic isolates), as described in Chiellini et al. [23].

### 2.5. Phenotypic Characterization of Bacterial Strains

#### 2.5.1. Bacterial Stress Resistance Assay

The bacterial resistance pattern toward a panel of five different stresses was assessed for each bacterial strain isolated from the seeds, three strains isolated from the GI samples, and three strains isolated from the GR samples, for a total of 30 tested strains. The five conditions were 0.0025% H_2_O_2_ (oxidative stress), 15% polyethylene glycol (PEG) 6000 (water potential stress), 2% NaCl (salt stress), 1 µg mL^−1^ streptomycin, and 5 µg mL^−1^ penicillin [24]; according to the authors [24], the two antibiotics were selected on the basis of the antibiotics commonly produced by rhizosphere microorganisms as indicators of biotic stress resistance. Each test was conducted in triplicate in Mueller Hinton Broth (MHB) in 96-well microplates in a total volume of 100 µL [25]. Both a positive control (the bacterial inoculum in MHB medium) and negative control (MHB medium) were set up. Bacterial growth was determined as the optical density at 600 nm (OD600) after 48 h of bacterial growth at 24 °C without shaking. The OD600 value measured for the positive control was considered as 100% growth, while the other measured values were reported as percentages of the growth in proportion to the positive control, according to Chiellini et al. [25]. A colorimetric code was used for data representation: growth values below 10% (red) correspond to the “absence of growth”, growth values between 10 and 50% (orange) correspond to “weak growth”, values between 50 and 75% (yellow) correspond to “growth”, and values greater than 75% (green) correspond to “complete growth”. The average values of three determinations were considered for the results and discussion.

#### 2.5.2. IAA Production

For all 30 bacterial strains tested for environmental stresses (see the previous paragraph), the IAA production was assessed in a liquid medium according to the protocol of the estimation by the Salkowski reagent. Briefly, 1 mL of each overnight liquid bacterial culture (continuous agitation at 24 °C), grown in TSB and supplemented with 1 mg mL^−1^ tryptophan, was centrifuged at 14,000 *g* for 5 min. Then, one volume of supernatant was mixed with two volumes of the Salkowski reagent and, after 30 min in the dark, the IAA production was measured at 530 nm [26] in a UV-1800 Spectrophotometer (Shimadzu, Kyoto, Japan). The quantification of the IAA produced was calculated through the construction of a standard curve using IAA at concentrations of 0, 1, 2, 5, and 10 µg mL^−1^ diluted in the culture medium of the bacterial strains. The quantification of the IAA produced by each strain was normalized on the basis of the number of bacterial cells calculated in each test. The number of bacterial cells was estimated through the OD600 measure. The results are expressed as mg of IAA produced by 1.5 × 10^8^ bacterial cells, corresponding to the McFarland standard n° 0.5 [27].

#### 2.5.3. Disk-Diffusion Assay of Cannabis Sativa Seed Extracts and Multivariate Statistical Analysis of Phenotypic Traits

The extracts, obtained as described in Section 2.1, were tested against all isolated bacterial strains for potential growth inhibitory effects. The agar disk-diffusion assay based on the Kirby–Bauer test [28] was used to test 10 µL of different concentrations of seed extract (10, 4, 2, and 1 mg mL^−1^), 10 µL of streptomycin (1 mg mL^−1^ solution, 10 µg total) used as a control, and 10 µL of 80% ethanol (Merck) used for seed extraction and dilution. Each substance was dropped on sterile 6 mm diameter Whatman paper disks that were placed on the surface of the agar plate inoculated with the bacterial culture diluted at an OD600 of 0.08–0.1, corresponding to McFarland standard n° 0.5 [27]. The test was conducted in triplicate on Mueller Hinton Agar (MHA) medium. The results were calculated as the average measure of the diameters of the halo surrounding each paper disk in the Petri dishes when the inhibition was present. The value of the ethanol inhibition (average of the three replicates) was subtracted from the average values obtained for each test at each concentration in order to normalize the data. The results were visualized with a heatmap constructed with the pheatmap package [29] in the R software [30]. In addition, a multivariate analysis with a principal component analysis (PCA) was performed to assess the relationships between multiple phenotypic characteristics of the strains, the isolation sources, and the taxonomy. The PCA was obtained using the princomp() function in the vegan R package [31]. Prior to the PCA, the data were scaled using a Z-score (e.g., each datum was centered by subtracting the column mean from each value in the column and scaled by dividing the centered values by the column standard deviation) transformation with the scale() function in the R base package.

### 2.6. Seed Germination Tests and Evaluation of Morphometric Parameters

**Figure 1:**
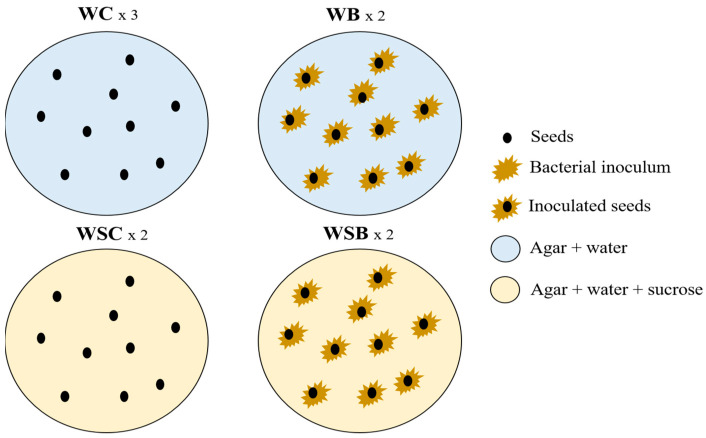
Schematic representation of the four treatments used for seed germination test.

The bacterial endophyte producing the highest amount of IAA was selected and used as an inoculum in different conditions for seed germination and growth. Two different media were prepared in sterility: a simple distilled water supplemented with 0.7% agar (W), and water supplemented with 0.7% agar and with 3% sucrose (WS). For each medium, we tested both the control without the bacterial inoculum (WC and WSC) and the bacterial inoculum (WB and WSB), for a total of four treatments. The tests were conducted in agar plates in sterility conditions for two weeks. For each agar plate, 10 seeds that were washed with sterile distilled water were used, for a total of 20 seeds (2 plates) for the treatments WSC, WB, and WSB and 30 seeds (three plates) for the WC treatment (Figure 1).

The bacterial inoculum for the WB and WSB treatments was obtained from an overnight liquid culture in TSB medium grown in agitation at 24 °C; the OD600 was measured to calculate the number of bacterial cells present in 1 mL of liquid culture (McFarland standard conversion). We diluted the inoculum with a sterile TSB medium in order to add 10 µL of inoculum, corresponding to about 3 × 10^7^ bacterial cells, to each seed. The inoculum was placed around each seed, on the agar surface under the biological flow, in order to guarantee sterility and avoid any effect of external bacterial contamination during seed germination and sprout growth (Figure 1). Plates with seeds were kept for two weeks in the growth chamber under a controlled temperature (24–22 °C). For the first three days of germination, seeds were kept in dark conditions as previously described [13], whereas from day 4 to day 14, seeds were kept under a 16/08 h day–night cycle with a PPFD of 70 µmol photons m^−1^ s^−1^. After two weeks, no external contamination was present on the surface of the agar plates, and in the WB and WSB treatment plates, in proximity to the inoculum that was added at the beginning of the experiment, a single bacterial morphology was present around the seeds.

On the two-week sprouts, the following morphometric parameters were evaluated: the number of germinated seeds, sprout fresh biomass, total sprout length, aerial part length, and root apparatus length. For the descriptive statistics, the average values with standard deviations were calculated considering all germinated sprouts in each treatment. For the analysis of variance (ANOVA), each sprout was considered as an independent statistical unit, and morphometric measurements were analyzed with respect to the “ADDITION” (i.e., addition or not of sucrose) and “STRAIN” (i.e., inoculum or not with the selected IAA-producing Can_S11 strain) categorical variables by two-way ANOVA (with an interaction term) using the *rstatix* package in R [32]. Additionally, we used the ggpubr package [33] to obtain a one-way ANOVA using the “TREATMENT” categorical variable (i.e., the combination of the ADDITION and STRAIN variables) and further compared the means of each treatment group with respect to control condition (e.g., class WC) using the *t*-test.

### 2.7. Nutraceutical Characteristics of Two-Week Sprouts of C. sativa

For each treatment, 1 g of pooled *C. sativa* sprouts in 10 mL of 80% ethanol were homogenized with an Ultraturrax (Kinematica Polytron PT MR 2100) and extracted overnight in the dark while shaking. After 20 min of centrifugation (Jouan CR 31 centrifuge, Newport Pagnell, UK) at 4500 g, the supernatant was collected, filtered (0.2 μm VWR International PBI, Milan, IT), and kept at −20 °C in the dark until use.

The *C. sativa* sprout extract was screened for the total polyphenol content as previously described in Chiellini et al. [34]. The total polyphenols were estimated as the Folin–Ciocalteu (FC) reducing capacity and expressed as mg of gallic acid equivalents per g on a fresh weight basis (mg GAE/g FW) of the *C. sativa* sprouts. The absorbance was recorded at 760 nm using a Perkin-Elmer Lambda 365 spectrophotometer (Perkin Elmer Italia, Milano, Italy).

The antioxidant capacity of *C. sativa* sprouts to reduce ferric iron (Fe^3+^) to ferrous iron (Fe^2+^) was determined by the FRAP assay as previously reported [35], and changes in the absorbance reflect the reducing power of the electron-donating antioxidants present in the sample. The FRAP assay was performed on the extract obtained as described above. The results were expressed as mg Fe^2+^/g FW using a water solution of FeSO_4_·7H_2_O (0.028–0.556 mg mL^−1^; VWR, Milan, Italy). The absorbance was measured at 593 nm using a Perkin-Elmer Lambda 365 spectrophotometer (Perkin Elmer Italia, Milano, Italy).

Analogously to the morphometric parameters, the results from the polyphenol quantification and FRAP assay were analyzed by one-way ANOVA with the “TREATMENT” categorical variable and by pairwise comparisons (*t*-test) with respect to the control conditions (e.g., class WC) using the ggpubr package [33].

## 3. Results

### 3.1. Molecular Characterization of the Isolated Strains and Phylogenetic Analysis

A total of 36 bacterial strains were isolated, 24 from the seeds and 12 from the two-week-old sprouts. The strains were divided into 12 different haplotypes. Eleven of those haplotypes were found in the seeds, while the strains isolated from the sprouts all belonged to the same haplotype. An NCBI blast analysis revealed that about 63.9% of the isolates (23 out of 36 strains) belonged to the *Firmicutes* phylum (Table 1) and are taxonomically related to *Staphylococcus* sp., *Bacillus* sp., *Psychrobacillus* sp., and *Paenibacillus* sp. genera (haplotypes A, C, E, G, M, and N). Two strains (haplotype F, 5.5%) were related to *Sphingomonas* sp. (*Alphaproteobacteria*), and three strains (haplotype D, 8.3%) were taxonomically close to the *Stenotrophomonas* sp. genus (*Gammaproteobacteria*). The remaining eight strains, representing 22.2% of the isolates (haplotypes B, H, I, and L), are taxonomically related to *Actinobacteria*, and in particular to the genera *Cellulomonas* sp., *Kocuria* sp., and *Curtobacterium* sp. The results of the BLAST analysis are shown in Table 1.

**Figure 2:**
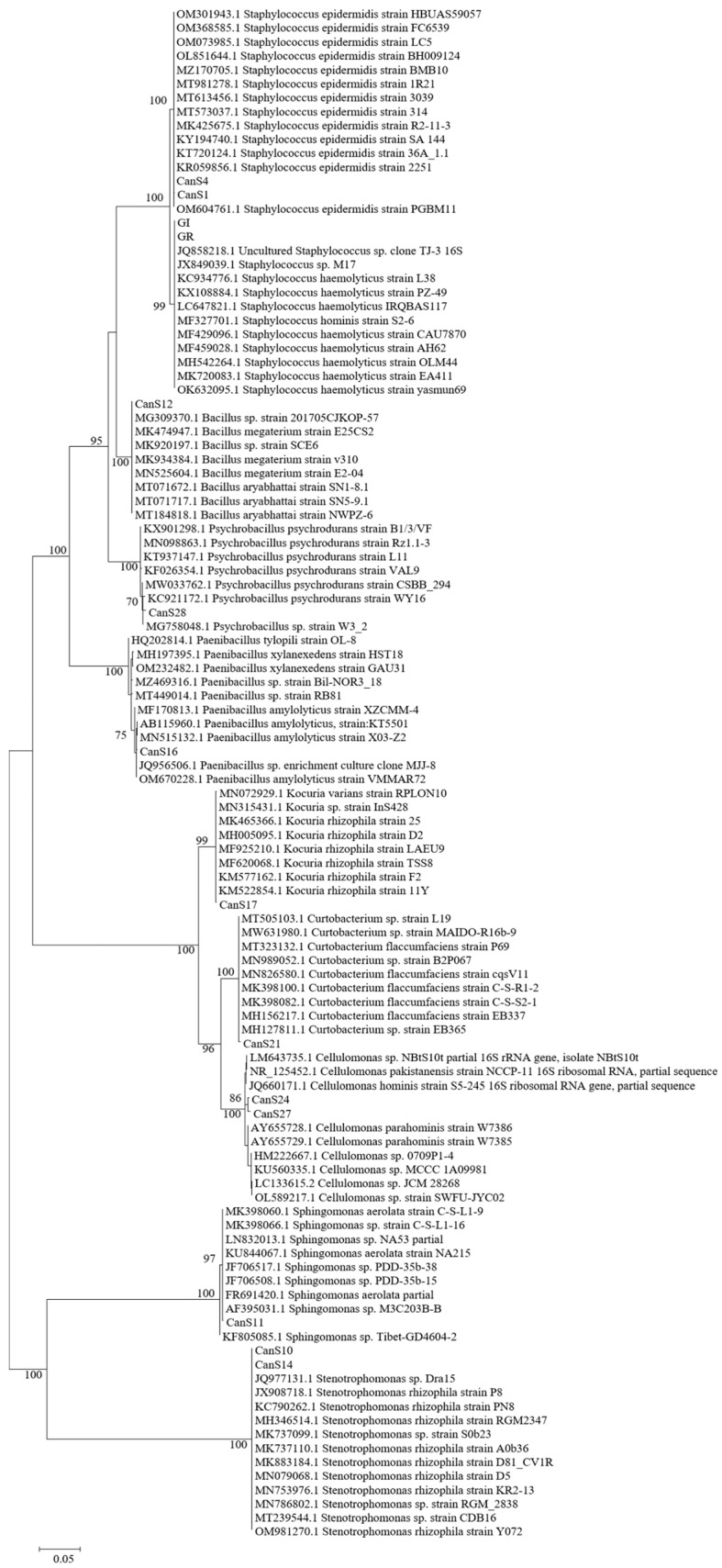
Phylogenetic tree reconstruction based on the 16S rRNA gene, obtained with maximum-likelihood method on a total of 110 sequences, 14 of them belonging to our strains and 96 high-quality sequences selected among those most similar to our isolated bacteria.

The phylogenetic analysis shown in Figure 2 allowed the recognition of the similarity at the species level for the bacterial strains.

The majority of the isolated strains belong to genus *Staphylococcus* sp. (Table 1), and, in detail, they are related to the species *S. epidermidis* (7 strains, haplotypes A and C) and *S. haemolyticus* (all 12 isolated strains isolated from the sprouts, haplotype N). The second most represented genus is *Cellulomonas* sp., including the four strains of haplotypes I and L (Table 1), for which it was not possible to perform a phylogenetic attribution with a high similarity and confidence to a described species. Indeed, in the clade in which such strains are comprised, there are different species such as *C. hominis*, *C. parahominis*, and *C. pakistaniensis* (Figure 2). Strains belonging to haplotype D are phylogenetically related to *Stenotrophomonas rhizophila*, while strains belonging to haplotype B are taxonomically attributed to species *Kocuria rhizophila*. Finally, the remaining strains isolated from seeds are phylogenetically closely related to *Bacillus* sp. (haplotype E), *Psychrobacillus psychrodurans* (haplotype M), *Paenibacillus* sp. (haplotype G), *Curtobacterium* sp (haplotype H), and *Sphingomonas* sp. (haplotype F). All the strains isolated from the two-week sprouts are not phylogenetically similar to any of the strains isolated from the seeds; according to the phylogenetic analysis, they are related to the species *Staphylococcus haemolyticus* (Figure 2).

### 3.2. Phenotypic Characterization of Isolated Bacterial Strains: IAA Production, Environmental Stresses, and Seed Extract Susceptibility

The IAA production, expressed as mg of IAA produced by 1.5 × 10^8^ bacterial cells, together with the results of the growth under stress conditions, expressed for each test as the % of growth with respect to the control (bacterial inoculum in MHB medium), are reported in Supplementary Table S1. According to our data, the highest IAA production was obtained by strain Can_S11 (2.1 mg of IAA), which is taxonomically related to the species *Sphingomonas areolata*, followed by strain Can_S28 (about 1.1 mg of IAA), which is related to *Psychrobacillus psychrodurans*, and the *Cellulomonas hominis*-related Can_S24 (about 0.88 mg of IAA). The strains showing no production of IAA are all related to the *Staphylococcus* sp. genus, in particular, all the *S. haemolyticus*-related strains isolated from the two-week-old sprouts and the *S. epidermidis*-related strains Can_S1, 4, 5, 6, and 8 (Supplementary Table S1).

**Figure 3:**
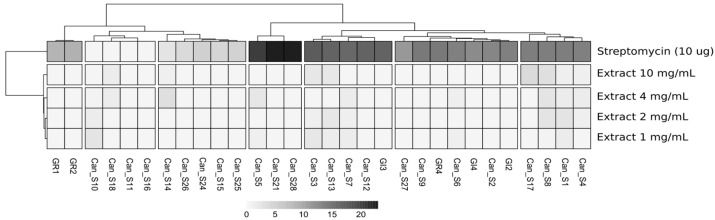
Heatmap showing the halo measures obtained after the exposure of the isolated strains to the seeds extract at four different concentrations, together with streptomycin. The values are the averaged measures of three replicates, normalized by removing the measures of the halos obtained with 80% EtOH, which was used as a solvent for seed extraction and dilution.

The seed extract susceptibility was assessed by testing four concentrations of seed extracts and measuring the values of the halos around the paper disk in the Kirby–Bauer disk diffusion test. The results are shown in Figure 3, while the raw measures of the halos are available in Supplementary Table S1.

According to the analysis, most of the strains are sensitive to 10 µg of streptomycin, with some exceptions represented by strains Can_S11 and Can_S18, both assigned to haplotype F and related to *Sphingomonas* sp., Can_S10 (*Stenotrophomonas rhizophyla*, haplotype D), and Can_S16 (*Paenibacillus amylolyticus,* haplotype G). The most sensitive strains are Can_S5, Can_S21, and Can_S28, respectively assigned to haplotypes C, M, and H. Considering the effect of the seed extract, the inhibition was almost absent in all strains at all tested concentrations, with only a few exceptions showing weak inhibition at the highest tested concentration, represented by strains Can_S1 (*Staphylococcus epidermidis* haplotype A), Can_S4 and 8 (*S. epidermidis* haplotype C), Can_S17 (haplotype B *Kocuria rhizophila*), Can S_13 (haplotype E, *Bacillus aryabhattai*), Can_S14 (*Stenotrophomonas rhizophila*, haplotype D), and Can_S18 (haplotype F, *Sphingomonas areolata*). The stress resistance patterns of the isolated strains, reported in Supplementary Table S1 (raw data) and Table 2, are represented with a colorimetric code indicating four levels of growth: red for the “absence of growth”, orange for “weak growth”, yellow for “moderate growth”, and green for “complete growth”.

According to stress tolerance patterns reported in Table 2, the most sensitive strain was Can_S13, which showed weak growth in presence of 15% PEG 6000, followed by the strain Can_S7, which showed complete growth only in the presence of 1 µg mL^−1^ streptomycin, and Can_S3, which showed weak growth in the presence of both streptomycin and PEG 6000. The strains showing higher resistance in the presence of the tested stresses were Can_S1, S2, S4, S6, and S8, which showed complete growth in presence of the antibiotics and 2% NaCl and weak growth with PEG 6000. All strains were totally sensitive to oxidative stress (H_2_O_2_), and only six strains (Can_S5, S9, S11, S12, S17, and S18) showed weak growth. Only four strains showed sensitivity to 1 μg mL^−1^ streptomycin, namely, Can_S13 (absence of growth), Can_S3 and GR4 (weak growth), and GR1 (growth).

**Figure 4:**
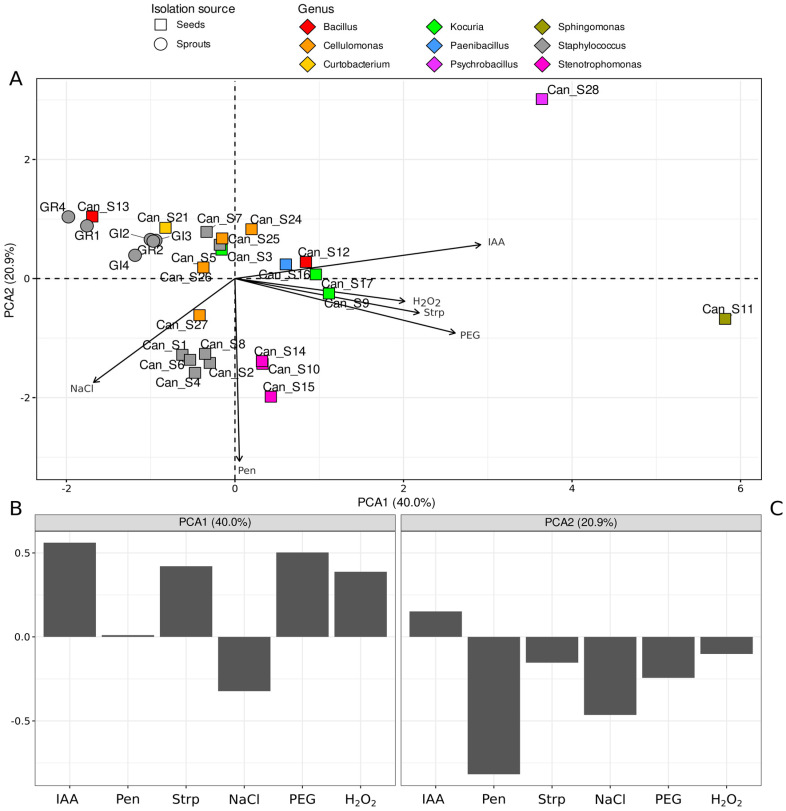
Principal component analysis representing the phenotypic characterization of bacterial strains. In this analysis, only variables from bacterial stress resistance and IAA production assays were considered. (**A**) Biplot of the ordination; the first (PC1) and second (PC2) principal components of the ordination analysis are reported. Each point represents an isolate, colored on the basis of its genus; circles represent isolates from sprouts, while squares represent isolates from seeds. Vectors point in the direction of highest variation of the variables and represent the contribution of each variable to a specific principal component (the greater the overlap with an axis, the greater the importance). (**B**,**C**) Importance of the different variables (e.g., variable loadings) on the first (**B**) and second (**C**) principal components.

The data from the stress tolerance evaluation and IAA production were further inspected by PCA. In this analysis, the original (scaled) variables were combined to form new variables, named principal components, with the aim of gradually explaining a higher proportion of dataset variance. The results of the analysis are reported in Figure 4A, representing the isolate ordination over the first two principal components (explaining 40.0% and 20.9% of the total variance, respectively) with vectors defining the direction of the changes of the original variables. To further inspect the contribution of the original variables to the principal components, variable loadings were reported (representative of the contribution of each original variable on the component) (Figure 4B,C).

The main differences were observed for the isolates Can_S11 and Can_S28 compared to all others, as they are clearly separated along the first component (PC1; the one explaining the higher proportion of variability) towards positive values. All other isolates displayed very similar stress resistance features and are closely grouped and overlapped at the origin of the graph. Nevertheless, we can observe a fair clusterization of the *Staphylococcus* strain isolated from sprouts (circles—towards negative values of the PC1 axis) compared to those isolated from seeds (squares—mainly found at negative values on the PC2 axis, with the exceptions of Can_S7 and Can_S5). All other strains showed stress resistance features that may be related to taxonomy (e.g., points with the same color are always close in the PCA ordination space). The grouping that can be observed on the PC2 axis seems to be mainly related to the higher resistance of a subset of strains (*Stenotrophomonas* and *Staphylococcus* isolated from seeds) to penicillin (5 µg mL^−1^) and to osmotic stress (NaCl 2%).

### 3.3. Seed Germination Assay in Presence of Different Treatment Conditions

According to the analysis performed on the isolated bacterial strains, especially regarding the production of IAA, we decided to use strain Can_S11 as a potential biostimulant for the preliminary germination tests in the four conditions defined in the Materials and Methods Section (Figure 1). The results of the germination test are summarized in Table 3. Both the morphometric parameters (% germinated seeds, fresh biomass, total length, root length, and aerial part length) and the nutraceutical characterization (polyphenols and FRAP) were evaluated and statistically analyzed.

In the treatments without Can_S11, about 70% of seeds were germinated, while in the presence of the inoculum, the germinated seeds were 60%. The average highest values of fresh biomass were measured in the sprouts grown in standard conditions (WC and WCS), while the lowest ones were observed in the presence of the bacterial inoculum (WB and WSB). The same trend was also observed for all the other parameters, namely, the total length of the sprouts, the root length, and the aerial part length.

Considering the nutraceutical parameters, the treatments showing the highest and the lowest polyphenol contents were represented by the bacterial inoculum in the control condition (WB, 2.693 ± 0.004 mg GAE/g FW) and in the presence of sucrose (WBS, 0.994 ± 0.055 mg GAE/g FW), respectively. The same trend was observed for the FRAP assay, with a maximum value of 2.821 ± 0.026 mg Fe^2+^/g FW for WB and a minimum value of 0.758 ± 0.023 mg Fe^2+^/g FW for the WSB treatment.

**Figure 5:**
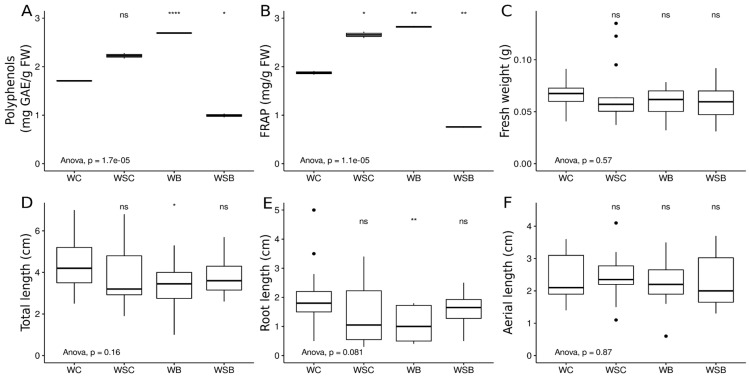
Boxplot representation of germination results of (**A**) polyphenols, (**B**) FRAP, (**C**) fresh weight, (**D**) total weight, (**E**) root length, and (**F**) aerial part length in each of the treatment groups (WC, without inoculum and no sucrose addition; WSC, without inoculum and sucrose addition; WB, with inoculum and no sucrose addition; WSB, with inoculum and sucrose addition). Results of the ANOVA comparison (*p*-value) between treatments is reported as notation below the boxplot. Results of statistical comparison, using *t*-test, of each treatment group with respect to the control (WC treatment) are reported as annotations above each boxplot (ns: not significant; * *p* < 0.05; ** *p* < 0.01; **** *p* < 0.0001).

The results of the morphometric characterization of sprouts were analyzed with the use of a two-way ANOVA performed on each of the four germination variables considered (e.g., fresh biomass, total sprout length, root length, and aerial part length) with respect to the categorical variables of “addition” (e.g., sucrose or none) and “strain” (e.g., presence or not of Can_S11 biostimulation). In all cases, the two-way ANOVA was not significant for the tested categorical variables nor for their interaction. Nevertheless, we found a significant difference for the total sprout length and root length when comparing the addition or not of the Can_S11 inoculum in absence of sucrose. To better visualize this difference, the germination results were further analyzed by comparing each combination of addition and strain (e.g., the “Treatment” variable) by means of ANOVA test and then by pairwise comparisons of each treatment group with the control treatment (WC) (Figure 5) using a *t*-test. As indicated by the notation above each boxplot in Figure 5, the WB treatment (the presence of the Can_S11 inoculum in the absence of sucrose addition) significantly reduced the total length and root length in sprouts with respect to the control (WC). In this last analysis, data from the quantification of polyphenols and the FRAP assay were additionally included. As shown in Figure 5, with respect to the basal condition (WC), a significantly higher content of total polyphenols was observed in sprouts under the WB treatment, whereas significantly lower values were obtained in the presence of the Can_S11 inoculum after sucrose addition (WSB treatment). A similar trend was also observed for the FRAP results, which highlighted significantly higher values in WB and WSC sprouts and significantly lower activity in the WSB sprouts with respect to the control sprouts.

## 4. Discussion

Bacteria isolated from seeds are dominated by Gram-positive strains, while Gram-negative isolates are mainly found in the early stages of seed development [36]. This is in agreement with our results since in our mature seeds we found all Gram-positive bacteria, except for the strains Can_S10, S14, and S15 (haplotype D) and Can_S11 and S18 (haplotype F). The internal tissues of the seeds represent a peculiar environment in which the conditions change as long as the maturation occurs; these changes might affect the bacterial community inhabiting it. Consequently, seed endophytic bacteria possess some characteristics that are not shared with endophytes from other tissues, such as the tolerance to osmotic pressure due to the accumulation of starch and the loss of water during the seed maturation process [36,37]. According to our data, 11 strains isolated from the seeds showed complete growth in presence of 2% NaCl, representing 45.8% of the 24 total isolated strains; moreover, all strains from the sprouts were tolerant to this stress condition as well. On the other hand, only six strains from the isolates were not tolerant to osmotic stress, enforcing the aforementioned hypothesis. In addition to the peculiar/selective seed environment, most of the endophytic bacterial population of the seeds probably has unknown cultivation conditions or is in a viable but non-cultivable state [7]. These aspects might (at least partially) explain the fact that all bacterial strains isolated from the seeds were not retrieved in the two-week-old sprouts. At the same time, we cannot exclude the possibility that the “enrichment” with TSB medium for 1 week, of both the disrupted and the non-disrupted sprouts (GI and GR), might have preferentially selected these bacteria belonging to the *S. haemolyticus* clade in the specific experimental conditions and that the selection and growth of this group of bacteria might have prevented the growth of the strain previously retrieved in the seeds.

Overall, by inspecting the loadings on the first dimension in the PCA analysis on the data from the stress tolerance evaluation and IAA production (Figure 4), we observed that strains Can_S11 and Can_S28 are characterized by higher IAA production and higher resistance to streptomycin as well as higher resistance to water potential stress (PEG 6000) and to oxidative stress (H_2_O_2_) but lower resistance to osmotic stress (NaCl). These two strains are different from all the others with respect to all the measured parameters. *Sphingomonas* strains have been widely described in past studies as endophytes with the ability to produce IAA and promote plant growth [38], even under stressful conditions for plants [39]. According to the literature, *Psychrobacillus psychrodurans* is also among the bacterial endophytes showing great potential as a plant-growth-promoting bacterium [40]. These data seem to be in agreement with our results regarding strains Can_S11 and Can_S28. Although we did not find elevated osmotic stress resistance in *Sphingomonas* sp. Can_S11 or in *Psychrobacillus* sp. Can_S28 (Table 2 and Figure 4), previous studies focused on the mangrove environment described *Psychrobacillus psychrodurans* species as an endophyte showing both PGP activity and high osmotic stress resistance [41] and *Sphingomonas* sp. as a halotolerant endophyte of *Salicornia europaea* L. [42].

Herein, the potential of the *Sphingomonas* sp. strain Can_S11 as a putative biostimulant has been preliminary investigated by the morphometric and nutraceutical analyses of the sprouts grown in the basal condition in the presence of Can_S11 inoculum (WB, Figure 1). Although the morphometric evaluation did not show any significant improvement in biomass or aerial part length, slight reductions in sprout root and total length were observed following the addition of *Sphingomonas* sp. Can_S11 inoculum. Moreover, the analyses of the total polyphenol content and the antioxidant activity, measured as the ferric reducing antioxidant power, clearly highlighted a positive effect of the application of *Sphingomonas* sp. Can_S11 on the nutraceutical and functional characteristics of *C. sativa* sprouts (Figure 5). Our results are in agreement with those observed by Almuhayawi et al. [43] on *Chenopodium* sprouts using an endophytic bacterium of the *Streptomyces* genus, isolated from the *Chenopodium* plant, as a biostimulant for sprout growth and functional properties; indeed, according to these authors, higher levels of polyphenols and flavonoids as well as increased antioxidant activities were found in *Chenopodium* sprouts after *Streptomyces* inoculation. Similarly, Briatia et al. [44] observed higher polyphenol and flavonoid contents in tartary buckwheat sprouts inoculated with *Herbaspirillum* spp., whereas higher antioxidant enzyme activities were detected in tomato (*Solanum lycopersicum* L.) seedlings inoculated with the endophytic bacterium *Pseudomonas* sp. TPs-04 [45]. Among others, bacteria endophytes can increase the contents of secondary metabolites through their ability to produce IAA, which is usually used by plants to produce sugar further used in secondary metabolite production, through a regulatory action on plant enzyme activities (e.g., phenylalanine ammonia lyase) and/or through the secretion of bacterial metabolites that can impact the production of some plant secondary metabolites. However, some variability might be observed depending on the growth conditions and/or plant species [43] (and references therein).

The addition of sucrose to the medium (WSC) also positively affected the polyphenol content and FRAP activity of *C. sativa* sprouts compared to the controls but did not impact any of the morphometric features analyzed here. These results are in line with what was observed by Wei et al. [46] in mung bean sprouts under sucrose treatment, highlighting increased contents of vitamin C and total phenolics as well as higher FRAP and DPPH scavenging rates. Surprisingly, the addition of both sucrose and bacterial inoculum to the *C. sativa* seeds did not interfere with the sprouts’ morphological features, whereas it negatively affected their nutraceutical profile, leading to significantly lower antioxidant activity and polyphenol levels than control sprouts (WC) and sprouts obtained in the presence of sucrose alone (WSC; *t*-test: *p* = 0.015 and *p* = 0.0052, respectively). These findings could be explained by the bacterial metabolism, as *Sphingomonas* was described as a sucrose-assimilating genus [47]; accordingly, other than consuming the sucrose in the medium, which is no longer available for the plant, it might produce some secondary metabolites that can interfere with the *C. sativa* seedling metabolism. Hence, further investigations will be addressed toward the biostimulant effect of strain Can_S11 in seeds and sprouts grown not only in vitro but also in soil.

According to previous works, the majority of cultivable bacterial endophytes of *C. sativa* were isolated from the plant tissues of adult plants (leaves and petioles) compared to the seeds; these bacterial strains were associated with the genera *Pantoea* sp., *Staphylococcus* sp., *Bacillus* sp., and *Enterobacter* sp. [16]. Our results are partially in agreement with these data since the *Staphylococcus* sp. and *Bacillus* sp. strains (haplotypes A, C, and E) represent 29.2% (7/24 strains) and 8.3% (2/24 strains), respectively, of all the bacteria isolated from the seeds. *Staphylococcus* sp. is a genus commonly associated with the endophytic communities of different plant species such as the medicinal plants *Echinacea* sp. [48] and *Chlorophytum borivilianum* [49], and it was also found in cotton [50], carrot [51], and soybean [52]. Interestingly, *Staphylococcus* species were also found as seed endophytes of maize [53,54]. Our data revealed that 19 isolates of a total of 36 were *Staphylococcus* sp., 7 of which were retrieved in the seeds and were closely related to *S. epidermidis* species, while 12 were isolated from the sprouts and were taxonomically related to *S. haemolyticus*. Reports of *S. epidermidis* and *S. haemolyticus* among plant-associated bacterial communities are available in the literature. *S. epidermidis* has been described as an endophyte of ginseng [55] and the *Anadenanthera colubrina* tree [56]. Chaudhry et al. [57] analyzed the genomes of diverse *S. epidermidis* strains of human pathogenic origin and from plants; the same authors recently highlighted the genomic plasticity of *S. epidermidis*, leading to its adaptability in diverse habitats and consequently making this species ecologically flexible [58]. The detection of *Staphylococcus haemolyticus*-related bacteria among endophytes is not surprising. Indeed, recent studies proved that this bacterial genus, despite being commonly known among human pathogenic bacteria, has been retrieved as a plant endophyte in different species such as willow [59,60] and the *Anadenanthera colubrina* tree [56] as well as in species of agricultural interest such as rice [58]. The clusterization of *Staphylococcus* sprout isolates (grey circles) from seed isolates (grey squares) towards negative values on the PC1 axis in the PCA analysis (Figure 4) suggests an overall different stress resistance of those strains (all identified as *Staphylococcus haemolyticus*) compared to other *Staphylococcus* isolated from seeds (identified as *Staphylococcus epidermidis*), with the exception of Can_S7 and Can_S5. Moreover, those isolates are mainly found at negative values on the PC2 axis, further suggesting a higher penicillin resistance with respect to sprout isolates.

Comparing the growth of the strains in the presence of streptomycin, both in the disk diffusion test assay (Figure 2, 10 µg total of the antibiotic substance) and in the stress resistance assay (Table 2, 0.1 µg of the antibiotic substance in 100 µL of the test volume), we can confirm that the four strains sensitive to the highest concentration tested in the disk diffusion assay (strains Can_S3, Can_S13, GR1, and GR4) also show a sensitivity at the lowest concentration tested in the latter assay. On the other hand, all the other strains that are sensitive to 10 µg of streptomycin are instead resistant to the lowest concentration of the substance. On the contrary, most of the strains seem to be sensitive to penicillin, as only 8 out of 36 (22.2%) strains showed growth or complete growth (yellow and green colors in Table 2). Since the use of 1 µg mL^−1^ streptomycin as well as 5 µg mL^−1^ penicillin is an indicator of biotic stress resistance and these two antibiotics are commonly produced by rhizospheric microorganisms [24], we can conclude that most of the isolated strains seem to be partially resistant to biotic stresses.

Despite a previous study showing that *C. sativa* L. seed extract has a selective inhibitory action against pathogenic bacterial strains as well as a potential role as a new antibiofilm agent [19], the extract did not show any inhibitory effect on the endophytes extracted from both the seeds and the sprouts. According to the recent literature, fungal endophytes are resistant to plant secondary metabolites that instead are toxic to pathogenic fungi [61] (and references therein). This evidence let us suppose that our bacterial endophytes might also be resistant to the seed secondary metabolites that previously showed an antimicrobial effect against pathogenic bacteria [19]. Considering that endophytic bacteria improve and/or induce the production of several secondary metabolites in plants and they share similar properties with plants, such as antimicrobial, anticancer, anti-inflammatory, and anti-HIV activities [62] (and references therein), we can conduct future studies for the investigation of the contribution of seed endophytic isolated strains to the antimicrobial and antibiofilm properties observed in the seed extracts.

## 5. Conclusions

Bacterial endophytes of *Cannabis sativa* L. cultivar Futura 75 were isolated from seeds and from two-week sprouts grown in vitro. Among the isolated strains, the phylogenetic characterization highlighted a dominance of *Firmicutes* (*Staphylococcus* sp.). The phenotypic characterization allowed us to identify two strains that were different from the others for their ability to produce IAA and for their resistance patterns toward abiotic and biotic stresses. One of these strains, the *Sphingomonas* sp. strain Can_S11 (*Alphaproteobacteria*), showed a potential ability to increase the nutraceutical properties of the *C. sativa* sprouts, particularly increasing the total polyphenol content and FRAP antioxidant activity. None of the isolated strains were susceptible to the seed extracts, which were previously successfully tested as antimicrobial and antibiofilm agents against human pathogenic bacteria, thus suggesting a possible defense/resistance mechanism asserted by the endophytes towards the host plant’s secondary metabolites. Future research will be addressed at completing the investigation of strain Can_S11 as a possible biostimulant, not only for *C. sativa* but also for other plant species.

## Figures and Tables

**Table 1:** Blast analysis results and ARDRA screening for the haplotype attribution of the isolated bacterial strains.

Haplotype	Strains	Sequenced Strain	Acc nr.	Length (bp)	Similarity 1° Blast and Affiliation	Similarity 1° Described Blast and Affiliation	Phylum
**A**	Can_S1; Can_S2	Can_S1	ON965345	1119		99.91% *Staphylococcus epidermidis* MK425675.1	*Firmicutes*
**B**	Can_S3; Can_S9; Can_S17	Can_S17	ON965346	1080		99.72% *Kocuria rhizophila* strain 25, MK465366.1	*Actinobacteria*
**C**	Can_S4; Can_S5; Can_S6; Can_S7; Can_S8	Can_S4	ON965347	1029		100% *Staphylococcus epidermidis* MT613456.1	*Firmicutes*
**D**	Can_S10; Can_S14; Can_S15	Can_S10	ON965348	1033	100% *Stenotrophomonas* sp. MT239544.1	100% *Stenotrophomonas rhizophila* strain KR2-13, MN753976.1	*Proteobacteria* (*Gamma*)
**D**	Can_S10; Can_S14; Can_S15	Can_S14	ON965349	1058		100% *Stenotrophomonas rhizophila* strain PN8 1, KC790262.1	*Proteobacteria* (*Gamma*)
**E**	Can_S12; Can_S13	Can_S12	ON965350	1059		100% *Bacillus aryabhattai* strain NWPZ-6, MT184818.1	*Firmicutes*
**F**	Can_S11; Can_S18	Can_S11	ON965351	1159	99.65% *Sphingomonas* sp. MK398066.1	99.65% *Sphingomonas aerolata* R-36940, FR691420.1	*Proteobacteria* (*Alpha*)
**G**	Can_S16	Can_S16	ON965352	1088		99.91% *Paenibacillus amylolyticus* strain VMSES13, OM963148.1	*Firmicutes*
**H**	Can_S21	Can_S21	ON965353	1063	99.53% *Curtobacterium* sp. strain MAIDO-R16b-9 MW631980.1	99.53% *Curtobacterium flaccumfaciens* strain Cff1037, CP041259.1	*Actinobacteria*
**I**	Can_S24; Can_S25; Can_S26	Can_S24	ON965354	1028	99.32% *Cellulomonas* sp. strain Y8, CP041203.1	99.22% *Cellulomonas hominis* strain S5-250 JQ660173.1	*Actinobacteria*
**L**	Can_S27	Can_S27	ON965355	1015	99.41% *Cellulomonas* sp. strain UYSB125, MT229317.1	99.31% *Cellulomonas hominis* strain S5-250, JQ660173.1	*Actinobacteria*
**M**	Can_S28	Can_S28	ON965356	1049	99.53% *Psychrobacillus* sp. strain W3_2, MG758048.1	99.43% *Psychrobacillus psychrodurans* strain CSBB_294, MW033762.1	*Firmicutes*
**N**	GI_1; GI_2; GI_3; GI_4; GI_5; GI_6	GI_2	ON965357	1046	100% Bacterium strain MTL7-8 16S, MH151274.1	100% *Staphylococcus haemolyticus* strain EA411, MK720083.1	*Firmicutes*
**N**	GR_1; GR_2; GR_3; GR_4; GR_5; GR_6;	GR_3	ON965358	1151	100% Bacterium strain MTL7-8 16S, MH151274.1	100% *Staphylococcus haemolyticus* strain OLM44, MH542264.1	*Firmicutes*

**Table 2:** Stress tolerance patterns against 5 µg mL^−1^ penicillin (pen), 1 µg mL^−1^ streptomycin (strp), 2% NaCl, 15% PEG 6000, and 0.0025% H_2_O_2_. Growth values below 10% (red color) correspond to the “absence of growth”, growth values between 10 and 50% (orange color) correspond to “weak growth”, values between 50 and 75% (yellow color) correspond to “growth”, and values greater than 75% (green color) correspond to “complete growth”. Strains are listed following the haplotype attribution.

Strains	Haplotype	Taxonomy	Pen 5 µg mL^−1^	Strp 1 µg mL^−1^	NaCl 2%	PEG 6000 15%	H_2_O_2_ 0.0025%
Can_S1	A	*Staphylococcus* sp.					
Can_S2	A	*Staphylococcus* sp.					
Can_S3	B	*Kocuria* sp.					
Can_S9	B	*Kocuria* sp.					
Can_S17	B	*Kocuria* sp.					
Can_S4	C	*Staphylococcus* sp.					
Can_S5	C	*Staphylococcus* sp.					
Can_S6	C	*Staphylococcus* sp.					
Can_S7	C	*Staphylococcus* sp.					
Can_S8	C	*Staphylococcus* sp.					
Can_S14	D	*Stenotrophomonas* sp.					
Can_S10	D	*Stenotrophomonas* sp.					
Can_S15	D	*Stenotrophomonas* sp.					
Can_S12	E	*Bacillus* sp.					
Can_S13	E	*Bacillus* sp.					
Can_S11	F	*Sphingomonas* sp.					
Can_S18	F	*Sphingomonas* sp.					
Can_S16	G	*Paenibacillus* sp.					
Can_S21	H	*Curtobacterium* sp.					
Can_S24	I	*Cellulomonas* sp.					
Can_S25	I	*Cellulomonas* sp.					
Can_S26	I	*Cellulomonas* sp.					
Can_S27	L	*Cellulomonas* sp.					
Can_S28	M	*Psychrobacillus* sp.					
GI2	N	*Staphylococcus* sp.					
GI3	N	*Staphylococcus* sp.					
GI4	N	*Staphylococcus* sp.					
GR1	N	*Staphylococcus* sp.					
GR2	N	*Staphylococcus* sp.					
GR4	N	*Staphylococcus* sp.					

**Table 3:** Parameters measured for the germination tests in the presence of four different treatments: % of germinated seeds; fresh biomass of the sprouts expressed as grams; the lengths of the sprouts, root apparatus, aerial part expressed in cm; polyphenols expressed as mg GAE/g FW; FRAP expressed as mg Fe^2+^/g FW. Values are the average ± standard deviation obtained as described in the Materials and Methods Section.

	% Seeds Germinated	Fresh Biomass	Total Length	Root Length	Aerial Part Length	Polyphenols	FRAP
WC	70%	0.067 ± 0.013	4.362 ± 1.178	1.943 ± 1.006	2.419 ± 0.698	1.707 ± 0.006	1.873 ± 0.055
WB	60%	0.059 ± 0.016	3.333 ± 1.184	1.116 ± 0.581	2.216 ± 0.732	2.693 ± 0.004	2.821 ± 0.026
WSC	70%	0.066 ± 0.03	3.893 ± 1.52	1.486 ± 1.073	2.407 ± 0.757	2.225 ± 0.08	2.655 ± 0.093
WSB	60%	0.059 ± 0.017	3.866 ± 0.982	1.575 ± 0.561	2.291 ± 0.842	0.994 ± 0.055	0.758 ± 0.023

## Data Availability

The 16S bacterial sequences obtained in this work are available in the NCBI public database under the accession numbers indicated in Table 1.

## Supplementary Materials

The following supporting information can be downloaded at: https://www.mdpi.com/article/10.3390/microorganisms10091742/s1, Table S1: Taxonomic and phenotypic characterization of the bacterial isolates. Columns indicate in order: strain name, haplotype, taxonomic attribution (NCBI), quantification of IAA production, results of the growth in presence of stress conditions, and row values of halos in disk diffusion assay against seed extract and solvent (EtOH 80%).
