# Supplementary material for: Characterization of the Cultivable Endophytic Bacterial Community of Seeds and Sprouts of Cannabis sativa L. and Perspectives for the Application as Biostimulants
Source: Microorganisms. 2022 Aug 29;10(9):1742. doi: 10.3390/microorganisms10091742 (PMC9506497; doi:10.3390/microorganisms10091742)
Supplement: Supplementary file 1 [file microorganisms-10-01742-s001.zip › microorganisms-1863289-supplementary.pdf]

Supplementary material

Table S1. Resume of the taxonomic and phenotypic characterization of the bacterial isolates. Columns indicate in order: Strain name, haplotype, Taxonomic attribution (NCBI), quantification of IAA production, results of the growth in presence of stress conditions, and row values of halos in disk diffusion assay against seeds extract and solvent (EtOH 80%).

					Results of the growth under stress conditions, expressed for each test as the % of growth with respect to the control (bacterial inoculum in MHB medium). The % values are calculated on the bases of the measured absorbances, as described in materials and methods.					Row values of halo measures (mm) in the disk diffusion assay against the seeds extracts. Tests showing no halo (complete resistance) are indicated with the number 6, that correspond to the diameter of the disk, and was used to normalize the data in the matrix used for the heatmap construction (Fig. 3)					
**Strain**	**Haplotype**	**Taxonomy**	**Isolation source**	**mg IAA/1.5 x 10^8 cells**	**Pen 5 µg mL^{-1}**	**Strp 1 µg mL^{-1}**	**NaCl 2%**	**PEG 6000 15%**	**H_2O_2 0.0025%**	**Strp (10 µg)**	**EtOH 80%**	**10 mg mL^{-1}**	**4 mg mL^{-1}**	**2 mg mL^{-1}**	**1 mg mL^{-1}**
Can_S1	A	Staphylococcus epidermidis	Seeds	0.005902	90.5	>100	>100	13.4	5.8	20	9	10	10.5	11.5	10.5
Can_S2	A	Staphylococcus epidermidis	Seeds	0.150492	>100	>100	76.7	19.9	3.9	19	10	7	9.5	8	9.5
Can_S3	B	Kocuria rhizophyla	Seeds	0.28623	9.5	31.1	6.7	48.7	6.8	23.5	7	9	7	8	9
Can_S4	C	Staphylococcus epidermidis	Seeds	0.032459	>100	>100	>100	15.7	5.1	19.5	9	10	10.5	10	8.5
Can_S5	C	Staphylococcus epidermidis	Seeds	0.059016	5.1	95.4	5.4	10.4	18.5	26.5	8	7	10	7	9
Can_S6	C	Staphylococcus epidermidis	Seeds	0.056066	98.9	99.5	82.5	16.6	4.7	19.5	10.5	10	11.5	10.5	11.5
Can_S7	C	Staphylococcus epidermidis	Seeds	0.315738	5.9	93.8	4.9	8.6	7.0	22.5	8.5	6	10	9.5	10
Can_S8	C	Staphylococcus epidermidis	Seeds	0.050164	83.6	93.3	>100	28.7	4.2	20	8	11	10.5	10.5	8.5
Can_S9	B	Kocuria rhizophyla	Seeds	0.197705	19.9	79.3	19.2	55.1	21.2	20.5	8	6	8	8	8
Can_S10	D	Stenotrophomonas rhizophyla	Seeds	0.224262	93.2	85.4	46.0	45.1	4.3	6	8.5	6	9	10	11
Can_S11	F	Sphingomonas areolata	Seeds	2.103934	32.8	>100	33.3	71.6	41.1	6	10	8	7	9.5	10.5

Can_S12	E	Bacillus aryabhattai	Seeds	0.165246	8.0	84.8	8.5	18.0	41.5	23	8.5	6	7	7.5	8
Can_S13	E	Bacillus aryabhattai	Seeds	0.129836	9.2	10.0	9.1	12.1	7.3	23	7	9	6	9	8.5
Can_S14	D	Stenotrophomonas rhizophyla	Seeds	0.227213	94.9	91.3	25.7	39.7	5.2	8.5	9	10	12	9	8
Can_S15	D	Stenotrophomonas rhizophyla	Seeds	0.247869	>100	95.2	53.0	42.7	4.3	10.5	12	7	10	10	10
Can_S16	G	Paenibacillus amylolyticus	Seeds	0.64623	13.5	>100	>100	19.7	10.0	6	6	6	6	6	6
Can_S17	B	Kocuria rhizophyla	Seeds	0.498689	20.6	88.2	18.5	39.2	15.0	20	8	12	8	7.5	7.5
Can_S18	F	Sphingomonas areolata	Seeds	nd	42.0	84.6	26.1	46.1	21.5	6	8.5	10	10	8	8
Can_S21	H	Curtobacterium flaccumfaciens	Seeds	0.33177	<0	90.6	87.7	6.0	<0	29	6	6	6	6	6
Can_S24	I	Cellulomonas hominis	Seeds	0.878168	<0	88.1	>100	19.3	<0	11.5	6	6	6	6	6
Can_S25	I	Cellulomonas hominis	Seeds	0.540105	<0	92.5	>100	20.3	<0	11	6	6	6	6	6
Can_S26	I	Cellulomonas hominis	Seeds	0.343681	20.3	96.2	>100	19.1	0.1	10	6	6	6	6	6
Can_S27	L	Cellulomonas hominis	Seeds	0.515476	75.2	98.2	>100	7.2	0.3	17.5	6	6	6	6	6
Can_S28	M	Psychrobacillus psychrodurans	Seeds	1.096118	<0	>100	<0	40.5	0.4	29	6	6	6	6	6
GI2	N	Staphylococcus haemolyticus	2-weeks sprouts	0.029405	0.1	99.3	90.6	7.7	0.7	19.5	8	7.7	7	7.7	7.3
GI3	N	Staphylococcus haemolyticus	2-weeks sprouts	0.057657	0.4	>100	>100	5.0	3.3	23	8.3	7.7	7	7	7
GI4	N	Staphylococcus haemolyticus	2-weeks sprouts	0.017038	17.4	90.1	94.1	4.8	2.1	18.5	9.7	7.3	7.7	8.3	8.3
GR1	N	Staphylococcus haemolyticus	2-weeks sprouts	0.003698	0.6	51.9	98.7	6.4	1.3	14.5	8.3	7.3	7	7	7.3
GR2	N	Staphylococcus haemolyticus	2-weeks sprouts	0.035271	1.2	98.1	92.7	7.4	2.3	15	8.7	7.7	7.7	7	8.3
GR4	N	Staphylococcus haemolyticus	2-weeks sprouts	0.035132	<0	32.6	87.4	6.6	1.2	20.5	8.3	7	7	7	7.3
